# Differences in biomass and silica content in typical plant communities with ecotones in the Min River estuary of southeast China

**DOI:** 10.7717/peerj.7218

**Published:** 2019-07-22

**Authors:** Hui Gao, Shuijing Zhai, Zhigao Sun, Juan Liu, Chuan Tong

**Affiliations:** 1 School of Geographical Sciences, Key Laboratory of Humid Subtropical Eco-geographical Process, Ministry of Education, Fujian Normal University, Fuzhou, China; 2 Innovation Center and Key Laboratory of Water Quality and Reservation in the Pearl River Delta, Institute of Environmental Research At Greater Bay, School of Environmental Science and Engineering, Guangzhou University, Guangzhou, China

**Keywords:** Variation, Silica, Ecotone, Competition, Marsh, Min River estuary

## Abstract

Silica (Si) is a basic nutrient requirement for many aquatic organisms and its biogeochemical cycle plays an important role in estuarine coastal ecosystems. However, little is known about the role Si plays during plant–plant interactive processes in the marsh ecosystems. Here, variations in biomass, biogenic silica (BSi) content, and available Si content of *Cyperus malaccensis*-dominated marshes, *Phragmites australis*-dominated marshes, and their ecotonal marshes were studied in the Shanyutan marsh in the Min River estuary, China. Results showed that *C. malaccensis* and *P. australis* biomass in ecotones was lower than those in typical communities by 46.4% and 46.3%, respectively. BSi content in aboveground organs of *C. malaccensis* and culms and roots of *P. australis* was lower in ecotones than in typical communities, whereas BSi content in other organs showed the opposite trend. Biomass allocation in *C. malaccensis* and *P. australis* roots in ecotones was higher by 56.9% and 19.5%, respectively, and BSi stock in *C. malaccensis* and *P. australis* roots was higher than that in typical communities by 120.9% and 18.9%, respectively. Available Si content in ecotonal marsh soils was 12.6% greater than that in typical communities. Thus, the two plant species may use different strategies for Si accumulation and allocation in ecotones to adapt to the competitive environment. *P. australis* may expand primarily via occupation of wider aboveground space, thereby increasing the Si accumulation capacity in aboveground organs. Meanwhile, *C. malaccensis* may increase the Si allocation capacity of its roots to withstand the pressure from *P. australis*. This study will provide new insights into marsh plant competition from the perspective of Si, which can also benefit plant management in marsh ecosystems.

## Introduction

Silica (Si) is considered as a beneficial element for plants (Ma & Yamaji, 2015; Klotzbücher et al., 2018; Coskun et al., 2019). Many terrestrial and aquatic plants accumulate high amounts of Si in their tissues (Struyf et al., 2007; Ma & Yamaji, 2015; Carey et al., 2017). Si is still not recognized as an essential element for plant growth; however, its beneficial effects on the development, yield, mineral nutrition, health, and survival have been observed in a variety of plant species for decades in agricultural ecosystems (Epstein, 1994; Datnoff, Deren & Snyder, 1997; Savant et al., 1999; Datnoff, Snyder & Korndörfer, 2001; Ma & Yamaji, 2006). During the last decade, Si has received increased attention in natural ecosystems owing to its importance in plant growth, nutrient content (Schaller et al., 2012b, 2013; Brackhage et al., 2013; Coskun et al., 2016), stoichiometry (Schaller & Struyf, 2013; Schaller et al., 2016; Schaller, Hines & Brackhage, 2017), providing rigidity to plant structures, and enhancing resistance to abiotic and biotic stresses (Epstein, 1994, 2009; Querné, Ragueneau & Poupart, 2012; Schoelynck et al., 2014). Si availability has a great influence on other nutrient content and the stoichiometry of marsh grasses during plant growth (Schaller et al., 2012b; Brackhage et al., 2013), and can influence element cycling during litter decomposition (Schaller, Hines & Brackhage, 2017). Furthermore, Si may affect the nutrient element turnover via altered plant nutrient and litter decomposition, and the trade-off between resource efficiency and plant stabilization/defense (Schaller, Brackhage & Dudel, 2012a; Schaller & Struyf, 2013; Schoelynck et al., 2014; Schaller et al., 2016). Si could play a significant role in vegetation dynamics, and the processing and functioning of ecosystems (Struyf & Conley, 2009; Schaller et al., 2012b; Schoelynck et al., 2014; Struyf et al., 2015; Carey et al., 2017; Coskun et al., 2019).

Estuarine marshes, located in the land–ocean transition zone, are some of the most sensitive ecosystems to global climate change (Simas, Nunes & Ferreira, 2001), and are considered as important areas for studying Si transport and processing (Struyf et al., 2007; Struyf & Conley, 2009; Carey & Fulweiler, 2013). The efficient use of Si by different plant species potentially influences competitiveness in dynamic environments (Struyf & Conley, 2009). Plants that are able to utilize Si to their benefit are favored under changing environmental conditions in estuaries at the community level (Querné, Ragueneau & Poupart, 2012). Si is important for plant decomposition processes, plant competitiveness, and stress tolerance (Schoelynck et al., 2014). Previous studies have been conducted to explore Si content variations of typical plant communities in estuarine marsh ecosystems (Struyf et al., 2005; Hou et al., 2010; Querné, Ragueneau & Poupart, 2012; Jacobs et al., 2013), whereas those in ecotones (i.e., the transitional zone between two plant species) remain neglected. The competition among different plant species and the ecological edge effect in ecotones are clearly obvious (Cadenasso et al., 2003; Theriot et al., 2013). However, Si cycling in ecotones and its role for plant competition in estuarine marshes has not been explained in recent studies (Schoelynck et al., 2014). As a beneficial nutrient, Si content might show more variability in ecotones compared to typical communities, and thus may provide new insight into marsh plant competition.

The Shanyutan marsh is the largest wetland in the Min River estuary in the transitional subtropical zone of southeast China. The main vegetation includes native species *Cyperus malaccensis, Phragmites australis*, and *C. compressus*, and invasive species *Spartina alterniflora*, which started to invade this marsh in 2002 (Tong et al., 2011). Different types of typical plant communities are distributed zonally from land to sea. The ecotones of two plant communities, that is, the transition regions gradually formed during the process of plant expansion, are clearly distinct in this zone. *C. malaccensis* and *P. australis* in ecotones grow as vigorously as those in typical communities. Therefore, this provides a suitable area to study the differences between ecotones and typical community zones in the Min River estuary. Studies regarding Si in the Min River estuary marshes have mainly focused on soil and porewater in typical vegetation areas (Zhai & Xue, 2016; Hou et al., 2017; Gao et al., 2018), whereas Si cycling in transitional systems has been neglected. Previous studies have shown that biomass allocation and biogenic silica (BSi) stock in roots was higher in ecotones than in typical communities (Gao et al., 2017). This has important implications for understanding the mechanisms behind habitat competition of different plant species. However, the accumulation and allocation of biomass and Si content among different plant species in ecotones of plant–plant interactive processes have been overlooked.

In the present study, the differences in biomass and BSi content of *P. australis* and *C. malaccensis* during their competition were investigated, as was the available Si content of soils in the Shanyutan marsh. The objectives were to: (i) compare the variation in Si content and biomass of typical communities with that of ecotones; and (ii) discuss Si allocation variations among plant organs and between typical communities and ecotones. This would not only enhance the knowledge regarding the variations in Si content, but also help understand the potential competitiveness mechanism of native plant species from Si cycling in the estuarine marsh ecosystems.

## Materials and Methods

### Study area

**Figure 1:**
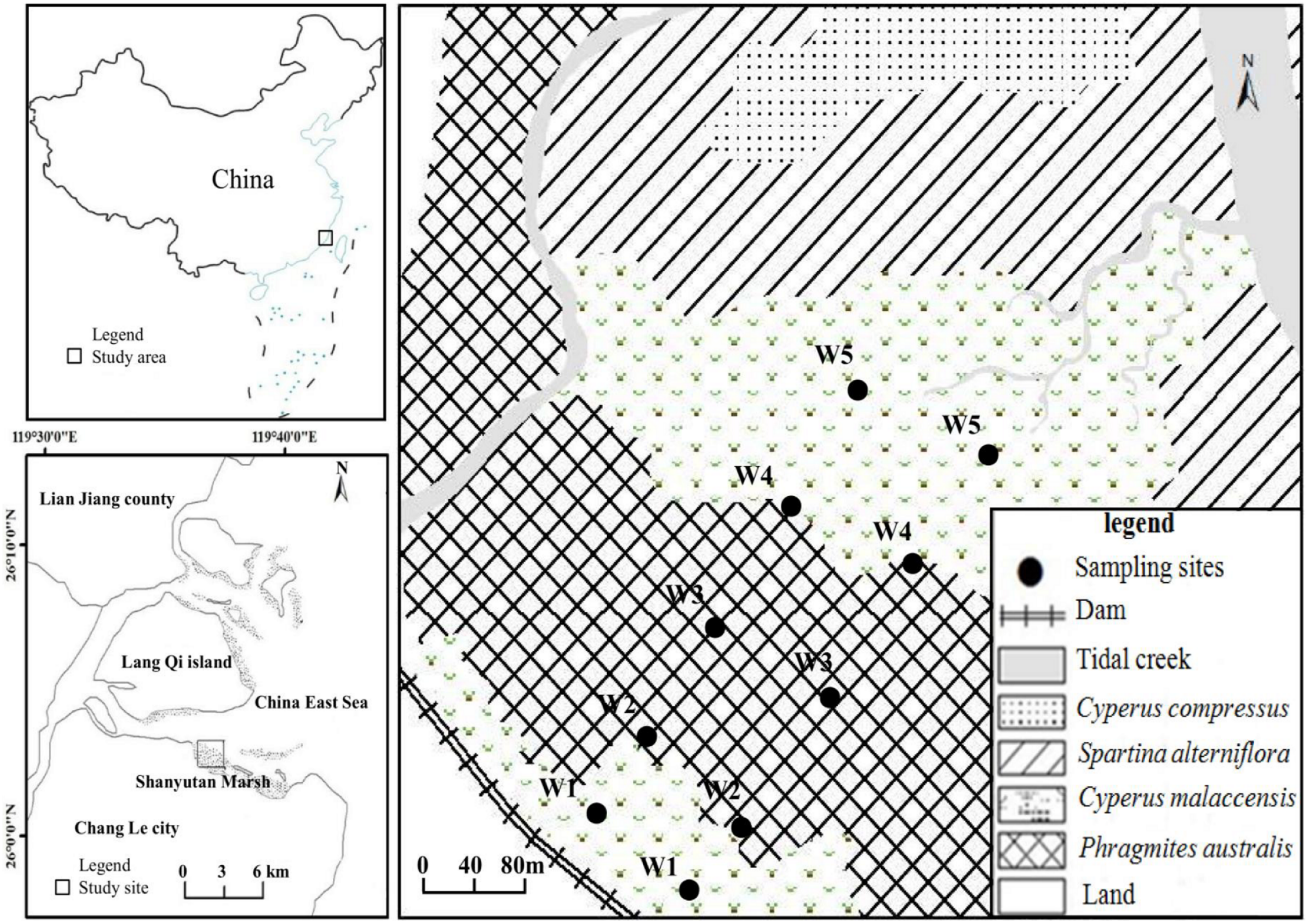
The study area and location of the sampling sites. W1, *Cyperus malaccensis* marsh; W2, *C. malaccensis*-*Phragmites australis* marsh; W3, *P. australis* marsh; W4, *P. australis*-*C. malaccensis* marsh; W5, *C. malaccensis* marsh.

The present study was undertaken in the Shanyutan marsh (119°34′12″–119°40′40″E, 26°00′36″–26°03′42″N), which is the largest wetland (approximately 3,120 ha) in the Min River estuary, southeast China (Liu, Zeng & Chen, 2006). The climate is relatively warm and wet, with a mean annual temperature of 19.6 °C and a mean annual precipitation of 1,300 mm. This area is featured by typical semi-diurnal tide with a mean tidal range of 4.37–4.46 m (Zhang, 1991; Dai, 2004). The marsh sediment is dominated by saline soil with low pH. The width of the Shanyutan coastal marsh (gradient 1–2°) is generally larger than one km (Liu, Zeng & Chen, 2006; Chen, Tang & Li, 2015). The main marsh vegetation includes three native species (*P. australis*, *C. malaccensis*, and *C. compressus*) and one invasive species *S. alterniflora* (Tong et al., 2010). In the western Shanyutan marsh, there are five typical plant communities distributed in a belt-like pattern from land to sea (Fig. 1): *C. malaccensis* community, *P. australis* community, *C. malaccensis* community, *S. alterniflora* community, and *C. compressus* community, with the latter two communities excluded from the present study. In this region, *P. australis* and *C. malaccensis* coexist without other species in the transitional zone, and are distributed from both *C. malaccensis* community to *P. australis* community and also from *P. australis* community to *C. malaccensis* community, with a width of approximately 50–100 m. Therefore, there were three typical communities and two distinct ecotones in our study.

### Sample collection and pretreatment

In July 2015, two parallel transects perpendicular to the coastline were laid from land to sea in the high-middle flat tidal zone of the western Shanyutan marsh. The distance between the two transects was approximately 100 m, and on each transect five sampling sites were selected that included three typical community marshes and two ecotonal marshes, that is, *C. malaccensis* community (closed to land and more than 50 m away from the dam, W1), *C. malaccensis*–*P. australis* community (*C. malaccensis* was dominant with a relative culms density of 82.7% per square meter, W2), *P. australis* community (W3), *P. australis*–*C. malaccensis* community (*C. malaccensis* was dominant with a relative culms density of 66.1% per square meter, W4), and *C. malaccensis* community (closed to sea, W5). To better distinguish the same plant species among the two ecotones, we recorded *C. malaccensis* in W2 as W2-C and *P. australis* as W2-P whereas *C. malaccensis* in W4 was recorded as W4-C and *P. australis* was W4-P.

Three replicate plots (50 × 50 cm) were randomly chosen from each of the five sites for the collection of soil and plant samples. Soil profiles (0–40 cm) were taken with a columnar sampler (diameter 10 cm and height 80 cm) and samples were collected at 10 cm intervals (0–10, 10–20, 20–30, and 30–40 cm) from each plot. Soil electrical conductivity and pH were measured simultaneously in situ using a portable instrument (Spectrum Technologies Inc., Chicago, IL, USA, and HACH-sensION3, Loveland, CO, USA, respectively). A total of 120 soil samples were collected and air-dried, ground, and sieved through an 80-mesh nylon sieve. Aboveground parts were clipped at the soil surface and separated into culms, leaves, and litter (withered parts). Underground parts were also collected. Plant samples of *C. malaccensis* or *P. australis* collected from typical communities or ecotones were wholly divided and pretreated based on the different organs of plant species in the ecotones. All plant organs were washed thoroughly with deionized water and dried in an oven (GZX-9140; MBE, Beijing, China) at 65 °C until a constant weight. Then, the samples were ground into a fine powder (<0.25 mm) for Si analysis.

### Sample analysis

To extract BSi, 30 mg of plant powder was digested for 5 h with Na_2_CO_3_ (0.1 mol/L) at 85 °C (Demaster, 1981; Struyf et al., 2005). BSi and available Si content of the soil were extracted with alkaline Na_2_CO_3_ (Demaster, 1981) and citric acid (Acquaye & Tinsley, 1965; Lu, 1999; Narayanaswamy & Prakash, 2010; Babu et al., 2016), respectively. The dissolved Si content in the extractions was measured using the molybdate blue spectrophotometric method. Total carbon and total nitrogen in soil were determined with a Vario EL Elemental Analyser (Elementar Scientific Instruments, Langenselbold, Germany). Soil grain size was measured with a Master Sizer 2000 Laser Particle Size Analyzer (Master Scientific Instruments, Malvern, UK). Soil organic matter (SOM) was analyzed by the K_2_Cr_2_O_7_ oxidation method. Soil bulk density (BD) was measured using the cutting ring method. Soil moisture content was the weight difference between fresh soils before and after drying in an oven at 105 °C for 24 h. The physical and chemical properties of soil profiles from the five sampling sites are shown in Table 1.

**Table 1:** Physical and chemical properties of soil profiles from five sampling sites.

Sites	EC (mS/cm)	pH	Moisture (%)	SOM (%)	BD (g/cm^3^)	TN (mg/g)	TC (mg/g)
W1	3.21 ± 0.86^ab^	6.43 ± 0.28^a^	39.78 ± 4.37^a^	4.38 ± 1.37^ab^	1.12 ± 0.11^a^	2.22 ± 1.06^a^	28.28 ± 11.63^a^
W2	3.77 ± 0.85^a^	6.28 ± 0.38^a^	42.23 ± 8.53^ac^	4.89 ± 0.92^ab^	1.06 ± 0.16^ac^	1.74 ± 0.70^ac^	22.12 ± 7.95^ac^
W3	3.55 ± 1.29^ab^	6.17 ± 0.27^ac^	50.98 ± 4.41^bd^	4.52 ± 1.03^ab^	0.94 ± 0.08^bd^	2.15 ± 0.64^ac^	26.46 ± 7.37^a^
W4	3.29 ± 0.90^ab^	5.96 ± 0.15^bc^	52.39 ± 3.15^b^	5.21 ± 1.33^a^	0.89 ± 0.05^b^	1.82 ± 0.09^ac^	22.12 ± 1.29^ac^
W5	2.77 ± 0.95^bc^	6.17 ± 0.32^ab^	46.99 ± 1.83^cd^	3.77 ± 1.09^b^	1.00 ± 0.03^cd^	1.52 ± 0.24^bc^	18.88 ± 2.67^bc^

**Notes:**

Values are means ± S.E. Different lowercase letters indicate statistical significant differences between sampling sites within the same column at the level of *p* < 0.05.

EC, electrical conductivity; Moisture, soil moisture; SOM, soil organic matter; BD, bulk density; TN, total nitrogen; TC, total carbon.

### Calculations

The biomass or BSi stock allocation proportions (AP, %) in different plant tissues were calculated by the following equation:

(1) $${\rm{AP}}\,{\rm{ = }}\,{{{\rm{Bi}}} \over {{\rm{Bn}}}}\, \times \,100\% $$

where, Bi (g/m^2^) is the biomass or BSi stock in different plant organs (including roots, culms, leaves, and litter); and Bn (g/m^2^) is the total biomass or total BSi stock. BSi stock is BSi content multiplied by relevant biomass (mg/m^2^).

The relative competition coefficient of *C. malaccensis* to *P. australis* (Rcp) was calculated based on De Wit’s (1960) model of resource competition to represent the interspecific competition ability as follows:

(2) $${\rm{Rcp}}\,{\rm{ = }}\,{{{\rm{Yce/Ype}}} \over {{\rm{Yct/Ypt}}}}$$

where, Yce (g/m^2^) and Yct (g/m^2^) are the biomass of *C. malaccensis* in ecotones and typical communities, respectively; and Ype (g/m^2^) and Ypt (g/m^2^) are the biomass of *P. australis* in ecotones and typical communities, respectively. Rcp > 1 means that *C. malaccensis* has a greater competition ability than that of *P. australis*.

For each plant species, we used the data regarding BSi content to calculate the values of roots/culms (*B_R_/B_C_*), roots/leaves (*B_R_/B_L_*), culms/leaves (*B_C_/B_L_*), and shoots/roots (*B_S_/B_R_*), which could represent the ability of relative transportation or immobilization of BSi under different situations, according to the methodology described by Dahmani-Muller et al. (2000). For example, if the value of *B_R_/B_C_* was lower, especially less than 1, more BSi was transported from roots to culms or more BSi was immobilized in culms.

Accumulation factors (AF) in different organs were calculated using the ratio of (Element)_plant_ to (Element)_soil_ (Duman, Cicek & Sezen, 2007) (Eq. (3)) to evaluate the ability of plants to absorb Si from marsh soils:

(3) $${\rm(AF)_{plant}} = {{{C_{{\rm{plant}}}}} \over {{C_{{\rm{soil}}}}}}$$

where, *C*_plant_ and *C*_soil_ (mg/g) were the average BSi content in different plant organs (including roots, culms, leaves, and litter) and in surface soils, respectively.

### Statistical analyses

Statistical analyses were performed with the SPSS Version 19.0 Statistical Software Package (SPSS Inc., Chicago, IL, USA). One-way analysis of variance was used to analyze significant differences in Si content of marsh plants and soils between typical communities and ecotones. Correlations between available Si content and physical and chemical properties were evaluated using Pearson correlation coefficients with a significance level of *p* = 0.05. Principal component analysis and stepwise linear regression analysis were used to test the major influencing factors of available Si content in marsh soils.

## Results

### Variation of biomass allocation of marsh plants in typical communities and ecotones

**Figure 2:**
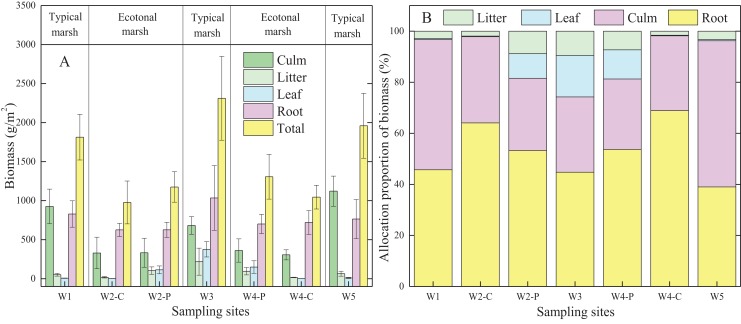
Biomass (A) and allocation proportion of biomass (B) to different organs of *Cyperus malaccensis* and *Phragmites australis* in typical communities and ecotones. The species *C. malaccensis* and *P. australis* in W2 were recorded as W2-C and W2-P while those in W4 were recorded as W4-C and W4-P respectively. Error bars represent standard error of the mean.

Biomass allocation was widely different in typical communities compared to ecotones for the same plant species (Fig. 2A; Table 2A). Each organ biomass of *C. malaccensis* and *P. australis* in typical communities was higher than that in ecotones. The total biomass of *C. malaccensis* and *P. australis* in typical communities was higher by 46.4% (*p* < 0.01) and 46.3% (*p* < 0.01) than those in ecotones, respectively.

**Table 2:** Biomass (A) and allocation proportion (B) to different organs of *Cyperus malaccensis* and *Phragmites australis* in typical communities and ecotones.

Species	Locations	Biomass (g/m^2^, A)					Allocation proportion (%, B)			
		Culm	Litter	Leaf	Root	Total	Culm	Litter	Leaf	Root
*Cyperus*	Typical	1,023.90 ± 97.89^***^	57.58 ± 6.17^***^	8.37 ± 1.55^***^	796.58 ± 32.52^*^	1,886.42 ± 73.10^***^	54.16^***^	3.04^**^	0.44^*^	42.36^***^
*malaccensis*	Ecotone	317.97 ± 11.69^***^	17.44 ± 1.05^***^	2.74 ± 0.07^***^	672.88 ± 47.17^*^	1,011.02 ± 34.36^***^	31.53^***^	1.73^**^	0.27^*^	66.47^***^
*Phragmites*	Typical	684.83 ± 46.08^***^	218.5 ± 70.64^*^	376.51 ± 39.78^***^	1,034.77 ± 168.95^**^	2,311.63 ± 219.01^***^	29.50^*^	9.45^*^	16.29^**^	44.76^**^
*australis*	Ecotone	346.49 ± 14.14^***^	99.39 ± 4.07^*^	131.23 ± 17.93^***^	663.58 ± 37.50^**^	1,240.69 ± 65.50^***^	27.95^*^	8.05^*^	10.53^**^	53.47^**^

**Note:**

Asterisks indicate significant differences between typical communities and ecotones within *Cyperus malaccensis* or *Phragmites australis* communities.

* *p* > 0.05.

** *p* < 0.05.

*** *p* < 0.01.

Generally, the allocation proportion to *C. malaccensis* root biomass was similar to that of *P. australis* in typical communities (*p* > 0.05); however, it was obviously higher in *C. malaccensis* than that in *P. australis* in the ecotones (*p* < 0.05; Fig. 2B; Table 2B). For the same plant species, root biomass allocation proportions in ecotones were higher than those in typical communities by 56.9% (*C. malaccensis*, *p* < 0.01) and 19.5% (*P. australis*, *p* < 0.05). However, biomass allocation proportions of culms (*p* < 0.01) and litter (*p* < 0.05) of *C. malaccensis* in ecotones were lower than those in typical communities by 41.8% and 43.1%, respectively, with no obvious discrepancy with *P. australis* (*p* > 0.05). The leaf biomass allocation proportion of *P. australis* in ecotones was significantly lower than that in typical communities (*p* < 0.05) by 35.4%, whereas no difference was observed for *C. malaccensis* (*p* > 0.05; Table 2B).

### BSi variation of marsh plants in typical communities and ecotones variation in BSi content

**Figure 3:**
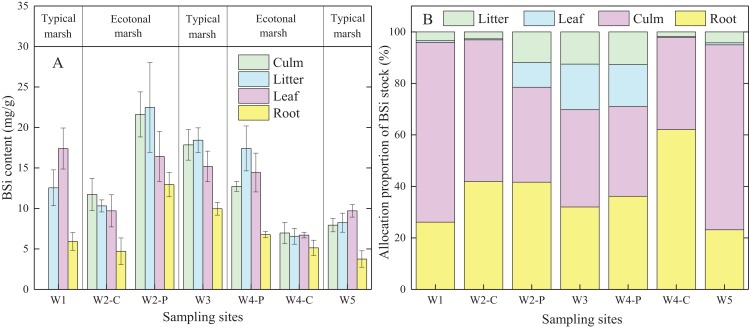
BSi content (A) and allocation proportion of BSi stock (B) of *Cyperus malaccensis* and *Phragmites australis* in typical communities and ecotones. The species *C. malaccensis* and *P. australis* in W2 were recorded as W2-C and W2-P while those in W4 were recorded as W4-C and W4-P respectively. Error bars represent standard error of the mean.

BSi content in different organs of *C. malaccensis* in typical communities and ecotones was lower than that of *P. australis* (*p* < 0.05; Fig. 3A; Table 3A). For *C. malaccensis*, the mean BSi content of different organs in W1, W2, W4, and W5 was 12.64, 9.90, 6.35, and 7.41 mg/g, respectively. The mean BSi content in *C. malaccensis* culms (*p* > 0.05), litter (*p* > 0.05), and leaves (*p* < 0.05) in ecotones was lower than that in typical communities, whereas the mean BSi content in roots was greater (by 2.27%, *p* > 0.05; Table 3A). The average BSi content of different organs in *P. australis* was highest in W2, followed by W3, and was lowest in W4 (Fig. 3A). The average BSi content of *P. australis* culms and roots in ecotones was lower than that in typical communities (*p* > 0.05), whereas the average BSi content in litter and leaves showed the opposite trend (*p* > 0.05; Table 3A). Thus, the same plant species had approximately more BSi near the shore and less BSi near the sea, except for *C. malaccensis* in W4.

**Table 3:** BSi content (A) and stock allocation proportion (B) to different organs of *Cyperus malaccensis* and *Phragmites australis* in typical communities and ecotones.

Species	Locations	BSi content (mg/g, A)				Allocation proportion (%, B)			
		Culm	Litter	Leaf	Root	Culm	Litter	Leaf	Root
*Cyperus*	Typical	11.06 ± 3.12^*^	10.40 ± 2.15^*^	13.55 ± 3.85^**^	4.84 ± 1.09^*^	71.54^*^	3.89^***^	0.71^*^	23.86^*^
*malaccensis*	Ecotone	9.35 ± 2.38^*^	8.44 ± 1.87^*^	8.21 ± 1.50^**^	4.95 ± 0.21^*^	44.71^*^	2.22^***^	0.35^*^	52.71^*^
*Phragmites*	Typical	17.86 ± 1.89^*^	18.43 ± 1.52^*^	15.19 ± 1.89^*^	9.98 ± 0.79^*^	38.54^*^	11.94^*^	17.33^*^	32.19^*^
*australis*	Ecotone	17.17 ± 4.45^*^	19.95 ± 2.53^*^	15.43 ± 0.99^*^	9.87 ± 3.09^*^	36.56^*^	13.70^*^	11.49^*^	38.26^*^

**Note:**

Asterisks indicate significant differences between typical communities and ecotones within *Cyperus malaccensis* or *Phragmites australis* communities.

* *p* > 0.05.

** *p* < 0.05.

*** *p* < 0.01.

### Allocation proportion of BSi stock

In ecotones, the allocation proportion of *P. australis* BSi stock was higher in roots and litter, and lower in leaves and culms than those in typical communities. The allocation proportion of BSi stock in *C. malaccensis* roots was higher in ecotones than in typical communities, whereas other organs showed the opposite trend (Fig. 3B). In contrast to BSi stocks in culms and leaves, the average allocation proportion in the roots of *C. malaccensis* and *P. australis* in ecotones was higher than that in typical communities by 120.9% and 18.9%, respectively (*p* > 0.05; Table 3B). For the different plant species, the allocation proportion of BSi stock in *C. malaccensis* roots (23.9%) was lower than that of *P. australis* (32.2%) in typical communities; however, it was higher in ecotones, with an allocation of 52.7% in *C. malaccensis* and 38.3% in *P. australis* (Table 3B). Therefore, both species might regulate BSi allocation in culms and leaves to be habituated to the homogeneous environment in typical communities while increasing the root BSi allocation to adapt to the competitive environmental conditions.

Variation in BSi transport was observed in *C. malaccensis* and *P. australis* as well as in typical communities and ecotones (Table 4A). The values of *B_R_/B_C_, B_R_/B_L_*, and *B_C_/B_L_* of *C. malaccensis* and *P. australis* in ecotones were higher than those in typical communities, whereas *B_S_/B_R_* ratios showed the opposite trend. Therefore, the mobility of BSi from roots to aboveground parts in ecotones was relatively weaker than that of typical communities.

**Table 4:** BSi transfer coefficients (A) and accumulation factors (B) in *Cyperus malaccensis* and *Phragmites australis* of typical communities and ecotones.

Species	Locations	Transfer coefficients (A)				Accumulation factors (B)			
		*B_R_/B_C_*	*B_R_/*B_*L*_	*B_C_/B_L_*	*B_S_/B_R_*	Culm	Litter	Leaf	Root
*Cyperus*	Typical	0.44	0.36	0.82	2.46	1.59	1.59	1.95	0.70
*malaccensis*	Ecotone	0.56	0.63	1.13	1.71	1.44	1.43	1.27	0.81
*Phragmites*	Typical	0.44	0.45	1.01	2.26	2.88	2.97	2.45	1.61
*australis*	Ecotone	0.54	0.61	1.11	1.92	2.48	3.07	2.23	1.35

The accumulation of BSi in different organs of the two plant species is shown in Table 4B. Apart from the roots, the AF of other organs of *C. malaccensis* in typical communities was generally higher than those in the ecotones. However, the AF in the roots of *P. australis* in the ecotones was lower than that in the typical communities by 16.2%.

### Variation in available Si content of marsh soils in typical communities and ecotones

**Figure 4:**
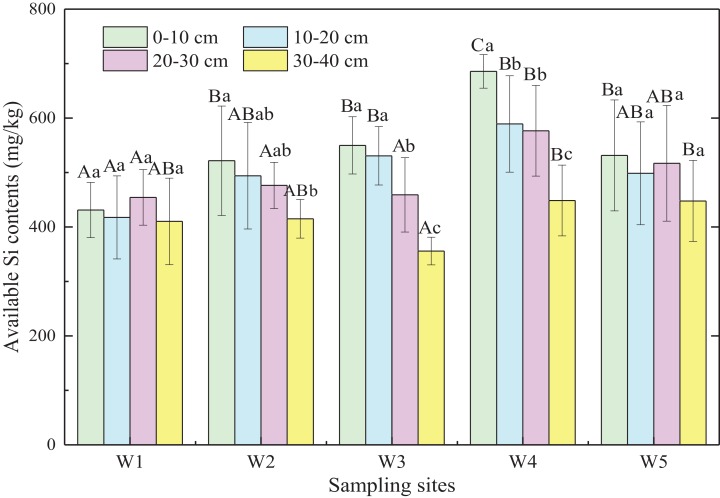
Available Si content of marsh soils in typical communities and ecotones. Error bars represent standard error of the mean. Uppercase letters represent significant differences (*p* < 0.05) in available Si content in the same soil layer between different sampling sites. Lowercase letters represent significant differences (*p* < 0.05) between different soil depths in the same site.

Generally, available Si content of marsh soils gradually increased from land to sea except for W5, and the mean value of available Si content in ecotones was significantly higher than that in typical communities by 12.6% (*p* < 0.05; Fig. 4). Significant differences were observed in available Si content of soil layers from 0 to 30 cm (*p* < 0.05) in the five sampling sites, with no significant difference in the 30–40 cm soil layer (*p* > 0.05). In general, available Si content decreased with increasing depth in different marshes (*p* < 0.01). Therefore, the available Si content in surface soil may be influenced by plant rooting, tides, and other factors, whereas the available Si content in deeper soils was relatively stable.

## Discussion

### Biomass allocation of marsh plants in ecotones and its implications for plant competition

Plasticity in biomass allocation determines the ability of plants to obtain resources in heterogeneous environments (Poorter, Remkes & Lambers, 1990), thereby influencing their competitiveness in different plant communities. Plants may improve their competitive ability by increasing biomass (such as roots, culms, etc.) and by allocating more resources to root systems under low nutrient conditions (Weiner, 2004; Wu, Chen & Du, 2010). In the Shanyutan marsh, the biomass of *C. malaccensis* and *P. australis* in ecotones was lower than that in typical communities (Table 2A), indicating that competition might exist between *C. malaccensis* and *P. australis* in ecotones, and plant growth was inhibited to some extent. Additionally, biomass allocation to *C. malaccensis* and *P. australis* roots in ecotones was higher than that in typical communities (Table 2B). In ecotones, the biomass allocation of *C. malaccensis* culms and *P. australis* leaves was significantly decreased. Therefore, plants might allocate more resources to roots, thus promoting root growth. To improve survival ability and competitiveness, plants may take different strategies for biomass allocation to make better use of resources under different environmental conditions (Lloret, Casanovas & Penuelas, 2010). In this region, both plant species might increase their ability to absorb soil nutrients, resulting in lower average C and N content of soils in ecotonal marshes (Table 1). Under this condition, plants in ecotones may increase the biomass allocation proportion of roots to improve their competitiveness and resist nutrient stress.

In ecotones, *P. australis* occupied more aboveground space with greater density and higher height, whereas the density of *C. malaccensis* was greatly reduced (Table S1). Thus, *C. malaccensis* had to promote a high biomass allocation proportion of roots to absorb more nutrients from subsurface layers and better withstand competition from *P. australis* in ecotones. In addition, the relative competitive stress of *C. malaccensis* was higher than that of *P. australis* (Rcp > 1), especially for underground resources (Table S2), which also showed that the underground parts of *C. malaccensis* had a stronger competition advantage in ecotones.

### Changed BSi stock allocation of marsh plants in ecotones and its implications for plant competition

Biomass of each organ in plants determines the amount of accumulated nutrients and the distribution of nutrients within plants (Zeng, Zhang & Tong, 2009; Li, 2013). Several studies have indicated that the differences of plant BSi storage were attributable to the discrepancies of biomass (Schoelynck et al., 2010; Struyf et al., 2015; Carey et al., 2017). In the present study, BSi stock allocation in *C. malaccensis* and *P. australis* roots was obviously higher in ecotones than that in typical communities, principally owing to the increased biomass allocation proportion (Fig. 2B; Table 2B). Especially for *C. malaccensis*, BSi allocation proportion of roots in ecotones almost doubled in typical communities, whereas the BSi allocation proportions in leaves and culms were nearly decreased by half. However, BSi allocation proportion in *P. australis* roots only increased by 18.9% in ecotones. Therefore, this might illustrate that *C. malaccensis* roots reserving more BSi may help it to compete with *P. australis* in ecotones.

Usually plants allocate more resources to roots to obtain more nutrients to improve their competitiveness under low nutrient conditions (Weiner, 2004). Regarding Si, active plant Si uptake has been suggested for native species exhibiting strong environmental stress conditions (Schoelynck & Struyf, 2016). The level of BSi accumulation in marsh plants could be automatically adaptive, that is, Si accumulation in the vegetation would be increased under stressful conditions (Schoelynck & Struyf, 2016). In the Shanyutan marsh, *P. australis* flourished easier to gain light and space on the ground than *C. malaccensis*. Therefore, *C. malaccensis* had to promote the Si enrichment ability of its roots to resist the spread of *P. australis* in ecotones, and adjust the absorption and allocation of Si to adapt to this competitive environment (Gao et al., 2017). In the present study, the allocation proportion of BSi stock in *C. malaccensis* and *P. australis* was different among ecotones, compared with that in the typical communities. In ecotones, BSi allocation proportion of each organ in *C. malaccensis* decreased except for the roots, whereas the increased value of BSi allocation proportion in *P. australis* was focused on the leaves and litter. Generally, Si has distinct effects in Si-accumulators (such as marsh plants) that are exposed to abiotic and biotic stresses (Ma & Yamaji, 2015; Coskun et al., 2019). Therefore, for superficial estimates, more BSi was focused on the roots in *C. malaccensis* rather than on the leaves in *P. australis* in ecotones to resist biotic pressure.

These results might indicate that the two plant species use different strategies for Si accumulation and allocation in ecotones to adapt to the competitive environment. *P. australis* expanded primarily by occupying a wider aboveground space and by increasing its Si accumulation capacity of aboveground organs, whereas *C. malaccensis* was able to resist the competitive pressure of *P. australis* by expanding the Si allocation of its roots.

### Differences of available Si content in marsh soils between typical communities and ecotones

Available Si content is important for measuring soil nutrient supply to plants (Babu et al., 2016; Klotzbücher et al., 2018). Pearson correlation analyses showed that significantly positive correlations occurred between available Si content and soil moisture and SOM (*p* < 0.01 or *p* < 0.05; Table S3). Available Si content was relatively higher in soils with more water content (Gao et al., 2015), which might be the reason for higher available Si content in ecotones (W2 and W4, Table 1). In addition, a previous study demonstrated that the decomposition of SOM could release Si, and soil organic acids and reductive conditions formed by organic matter degradation may damage iron-Si complexes, which are also conducive to Si dissolution (Ma, Chen & Chu, 2016). The higher available Si content observed in ecotonal marsh soils might also be related to the higher SOM (Table 1) compared to typical communities. Thus, soil moisture and SOM had important effects on the distribution of soil available Si in the Shanyutan marsh.

Principal component analyses showed that the major factors influencing available Si content were soil BD, grain composition, pH, and organic components (a variance contribution rate of 81.0%; Table S4). Furthermore, stepwise linear regression analysis revealed that soil BD was the crucial factor influencing available Si content (available Si = −301.691 × BD + 793.286, *p* = 0.006). In the present study, available Si content was relatively higher in ecotones than in typical communities and had a significantly negative correlation with BD (*p* < 0.01; Table S3), which might be due to the root systems of the vegetation. Previous studies indicated that root growth was limited because of the hardness and lack of oxygen supply in soils with higher BD, thus decreasing root dry weight (Grzesiak, 2009; Singh, Salaria & Kaul, 2015). In the present study, the mean biomass allocation to root systems in ecotones (60.0%) was higher than that in typical communities (43.56%, *p* < 0.05; Table 2B), whereas soil BD showed the opposite trend in ecotones (0.98 g/cm^3^) and typical communities (1.02 g/cm^3^, *p* > 0.05). Therefore, this might result in the higher available Si content in the marsh soils of ecotones.

## Conclusions

The biomass and BSi stock allocation proportion of *C. malaccensis* and *P. australis* roots were higher in ecotones than those in typical communities in the Shanyutan marsh. In ecotones, *P. australis* might expand primarily by occupying a wider aboveground space and increasing the Si accumulation capacity of aboveground organs, whereas *C. malaccensis* might resist the competition pressure of *P. australis* by increasing the Si allocation capacity of its roots. Available Si content in ecotone soils was also higher than that in typical community soils. Soil moisture, SOM, BD, and plant assimilation were the major factors influencing available Si content in the Shanyutan marsh.

## Supplemental Information

10.7717/peerj.7218/supp-1 Supplemental Information 1 Raw data applied for data analyses and preparation for all Figures and Tables. Biogenic Si content, biomass, density and height, available Si content, and soil properties.

10.7717/peerj.7218/supp-2 Supplemental Information 2 Mean values of height and density of marsh plants in typical communities and ecotones. Note: Asterisks indicate significant differences between typical communities and ecotones within *Cyperus malaccensis* or *Phragmites australis* communities (^*^ means *p* > 0.05, ^**^ means *p* < 0.05, and ^***^ means *p* < 0.01).

10.7717/peerj.7218/supp-3 Supplemental Information 3 Relative competition coefficient of *C. malaccensis* to *P. australis* in ecotones.

10.7717/peerj.7218/supp-4 Supplemental Information 4 Correlation coefficients between available Si contents and physical and chemical properties in marsh soils. Note: Asterisks indicate significant correlations between available Si contents and physical and chemical properties in marsh soils (^*^ means *p* < 0.05, ^**^ means *p* < 0.01).

10.7717/peerj.7218/supp-5 Supplemental Information 5 Eigenvalues and rotated component matrix of available Si content in soils of the Shanyutan marsh.

## Additional Information and Declarations

### Data Availability

The following information was supplied regarding data availability: Data are available as a Supplemental File.
