# Supplementary material for: Differences in biomass and silica content in typical plant communities with ecotones in the Min River estuary of southeast China
Source: PeerJ. 2019 Jul 22;7:e7218. doi: 10.7717/peerj.7218 (PMC6657677; doi:10.7717/peerj.7218)
Supplement: Supplemental Information 2 — Note: Asterisks indicate significant differences between typical communities and ecotones within Cyperus malaccensis or Phragmites australis communities (* means p > 0.05, ** means p < 0.05, and *** means p < 0.01). [file peerj-07-7218-s002.doc]

| Species | Height (cm) | | Density (individual plants/m^2^) | |
| --- | --- | --- | --- | --- |
|  | Typical | Ecotone | Typical | Ecotone |
| *Cyperus malaccensis* | 110.79±3.68^*^ | 110.75±3.43^*^ | 1286.34±165.54^***^ | 421.00±93.81^***^ |
| *Phragmites australis* | 181.77±10.91^***^ | 122.50±4.67^***^ | 80.00±7.52^**^ | 126.00±19.30^**^ |
