# Supplementary material for: Differences in biomass and silica content in typical plant communities with ecotones in the Min River estuary of southeast China
Source: PeerJ. 2019 Jul 22;7:e7218. doi: 10.7717/peerj.7218 (PMC6657677; doi:10.7717/peerj.7218)
Supplement: Supplemental Information 3 [file peerj-07-7218-s003.doc]

| Ecotones | Belowground | Aboveground | The whole plant |
| --- | --- | --- | --- |
| W2 | 3.61 | 2.40 | 3.07 |
| W4 | 13.05 | 5.39 | 8.86 |
