# Supplementary material for: Differences in biomass and silica content in typical plant communities with ecotones in the Min River estuary of southeast China
Source: PeerJ. 2019 Jul 22;7:e7218. doi: 10.7717/peerj.7218 (PMC6657677; doi:10.7717/peerj.7218)
Supplement: Supplemental Information 4 — Note: Asterisks indicate significant correlations between available Si contents and physical and chemical properties in marsh soils (* means p < 0.05, ** means p < 0.01). [file peerj-07-7218-s004.doc]

| Variables | Moisture | BD | SOM | Clay | Silt | Sand | Available Si |
| --- | --- | --- | --- | --- | --- | --- | --- |
| Moisture | 1 |  |  |  |  |  |  |
| BD | -0.959^**^ | 1 |  |  |  |  |  |
| SOM | 0.252 | -0.387^**^ | 1 |  |  |  |  |
| Clay | -0.026 | 0.076 | -0.056 | 1 |  |  |  |
| Silt | -0.178 | 0.189 | -0.075 | 0.557^**^ | 1 |  |  |
| Sand | 0.149 | -0.173 | 0.077 | -0.757^**^ | -0.964^**^ | 1 |  |
| Available Si | 0.444^**^ | -0.430^**^ | 0.309^*^ | -0.209 | -0.366 | 0.354 | 1 |
