# Supplementary material for: Differences in biomass and silica content in typical plant communities with ecotones in the Min River estuary of southeast China
Source: PeerJ. 2019 Jul 22;7:e7218. doi: 10.7717/peerj.7218 (PMC6657677; doi:10.7717/peerj.7218)
Supplement: Supplemental Information 5 [file peerj-07-7218-s005.doc]

| Components | Initial Eigenvalues | | | Variables | Components | | | |
| --- | --- | --- | --- | --- | --- | --- | --- | --- |
|  | Total | Variance (%) | Cumulative (%) |  | Z_1_ | Z_2_ | Z_3_ | Z_4_ |
| 1 | 3.798 | 34.527 | 34.527 | Moisture | 0.692 | 0.344 | -0.464 | -0.263 |
| 2 | 2.572 | 23.380 | 57.907 | BD | -0.747 | -0.393 | 0.397 | 0.142 |
| 3 | 1.532 | 13.924 | 71.831 | EC | -0.575 | 0.343 | -0.164 | 0.506 |
| 4 | 0.011 | 9.192 | 81.022 | pH | -0.209 | 0.137 | 0.587 | -0.376 |
| 5 | 0.750 | 6.814 | 87.836 | SOM | 0.470 | 0.413 | -0.028 | 0.601 |
| 6 | 0.736 | 6.689 | 94.525 | Clay | -0.411 | 0.655 | -0.070 | -0.368 |
| 7 | 0.309 | 2.813 | 97.338 | Silt | -0.705 | 0.567 | -0.278 | 0.011 |
| 8 | 0.260 | 2.362 | 99.699 | Sand | 0.686 | -0.654 | 0.241 | 0.108 |
| 9 | 0.026 | 0.235 | 99.934 | TN | 0.612 | 0.609 | 0.435 | 0.033 |
| 10 | 0.007 | 0.066 | 100.000 | TC | 0.558 | 0.638 | 0.492 | 0.068 |
